# The genetic diversity of “papillomavirome” in bovine teat papilloma lesions

**DOI:** 10.1186/s42523-021-00114-3

**Published:** 2021-07-28

**Authors:** Jéssica Tatiane Sauthier, Cíntia Daudt, Flavio Roberto Chaves da Silva, Christian Diniz Beduschi Travassos Alves, Fabiana Quoos Mayer, Ronaldo Michel Bianchi, David Driemeier, Rodrigo Silva Araujo Streit, Charley Christian Staats, Cláudio Wageck Canal, Matheus Nunes Weber

**Affiliations:** 1 Laboratório de Virologia Veterinária, Faculdade de Veterinária, Universidade Federal do Rio Grande do Sul (UFRGS), Porto Alegre, RS Brazil; 2 Laboratório de Virologia Geral eParasitologia, Centro de Ciências Biológicas e da Natureza, Universidade Federal do Acre, Rio Branco, AC Brazil; 3 Laboratório de Biologia Molecular, Instituto de Pesquisas Veterinárias Desidério Finamor, Fundação Estadual de Pesquisa Agropecuária, Eldorado do Sul, RS Brazil; 4 Setor de Patologia Veterinária, Faculdade de Veterinária, Universidade Federal do Rio Grande do Sul (UFRGS), Porto Alegre, RS Brazil; 5 Centro de Biotecnologia, Universidade Federal do Rio Grande do Sul (UFRGS), Porto Alegre, RS Brazil; 6 Laboratório de Microbiologia Molecular, Instituto de Ciências da Saúde, Universidade Feevale, Novo Hamburgo, RS Brazil

**Keywords:** BPV, Rolling-circle amplification, High-throughput sequencing, Cattle

## Abstract

**Background:**

Papillomaviruses are small nonenveloped, circular double-stranded DNA viruses that belong to the *Papillomaviridae* family. To date, 29 *Bos taurus papillomavirus* (BPV) types have been described. Studies involving mixed BPV infections have rarely been reported in contrast to human papillomavirus (HPV), which is commonly described in numerous studies showing coinfections. Moreover, previous studies had shown that HPV coinfections increase the risk of carcinogenesis. In the present study, we used rolling-circle amplification followed by a high-throughput sequencing (RCA-HTS) approach in 23 teat papillomas from southern Brazil.

**Results:**

Eleven well-characterized BPV types and 14 putative new BPV types were genetically characterized into the *Xi*, *Epsilon* and *Dyoxipapillomavirus* genera according to phylogenetic analysis of the L1 gene, which expands the previous 29 BPV types to 43. Moreover, BPV coinfections were detected in the majority (56.3%) of the papilloma lesions analyzed, suggesting a genetic diverse “papillomavirome” in bovine teat warts.

**Conclusions:**

The data generated in this study support the possibility that a wide range of BPV is probably underdetected by conventional molecular detection tools, and that BPV coinfections are underestimated and probably genetic diverse. Additionally, 14 new BPV types were characterized, increasing the knowledge regarding BPV genetic diversity.

**Supplementary Information:**

The online version contains supplementary material available at 10.1186/s42523-021-00114-3.

## Background

Papillomaviruses (PVs) are small nonenveloped, circular double-stranded DNA viruses of approximately 8 kb in length belonging to the *Papillomaviridae* family [1–3]. PVs were recently classified into the order *Zurhausenvirales*, class *Papovaviricets,* phylum *Cossaviricota,* kingdom *Shotokuvirae* and realm *Monodnaviria* [4]. These viruses infect a wide range of animals and are well-known etiological agents of skin warts and neoplasias in their hosts [5, 6].

*Bos taurus papillomavirus* (BPV) induces teat papillomatosis, a worldwide problem that can result in economic losses by predisposing dairy cows to secondary infections and decreasing milk production [7, 8]. Teat papillomatosis results in proliferative lesions affecting the stratified squamous epithelium of the teat skin [9, 10].

Currently, 29 BPV types are characterized and classified into five genera: *Delta* (BPV1, 2, 13 and 14), *Xi* (BPV3, 4, 6, 9, 10, 11, 12, 15, 17, 20, 23, 24, 26, 28 and 29), *Epsilon* (BPV5, 8 and 25), *Dyoxi* (BPV7) and *Dyokappapapillomavirus* (BPV16, 18 and 22). In addition, BPV19, 21 and 27 belong to the unclassified genus. Comparatively, approximately 225 human papillomaviruses (HPVs) have been described and fully characterized [11] where some of them, i.e. HPV16, are potently associated with cervical cancer [12]. Furthermore, in the last decade, a large number of studies have shown coinfections with different HPV types. Some of these studies used high throughput sequencing (HTS) [13], revealing that infections with multiple HPV types are common and are associated with an increased risk of carcinogenesis [14, 15], mainly in cervical cancer [14, 16–18]. The combination of HPV types, and the presence of high-risk (HR) HPV (i.e. 16—the most potent type—, 18, 31, 33, 35, 39, 45, 51, 52, 56, 58, and 59) also act in increase of carcinogenicity to humans [19].

Although studies with mixed BPV infections are rarely reported, coinfections of several new and putative types and classical BPVs have been described in bovine papillomatous lesions [20–24]. However, these studies only evaluated a limited number of animals [21, 23, 24]. Unlike conventional PCR with specific degenerate FAP primers [25] followed by Sanger sequencing, HTS has no need for specific primers for a specific target. When combined with the enrichment of circular DNA using RCA, this method offers a robust alternative to identify BPV types that other techniques lack sensitivity to [23].

Herein, we described the analysis of teat papillomatous samples using rolling circle amplification (RCA) followed by high-throughput sequencing (HTS) to detect PV types and increase the knowledge about BPV diversity and coinfections.

## Results

### Overview

**Fig. 1:**
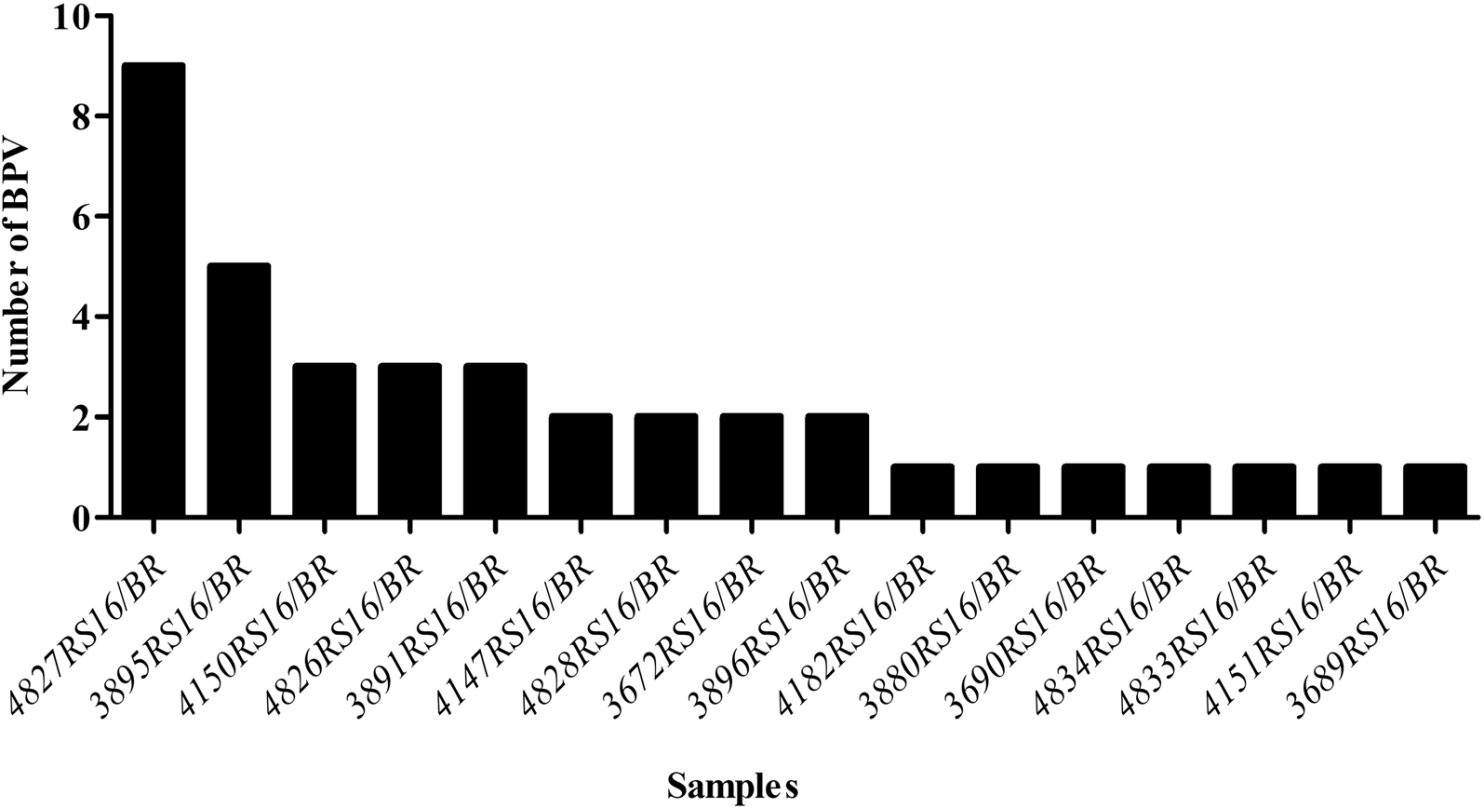
Diversity of bovine papillomavirus (BPV) present in the samples of this study based on the presence of complete and partial (length > 350 bp) L1 gene sequences. BPV types, putative new BPV types and new BPV types described in this study were included

Teat wart samples from 23 dairy cows were analyzed by employing RCA-HTS to investigate genetic PV diversity and coinfections. Sixteen out of the 23 samples contained PV-related sequences (Fig. 1). The raw number of high-quality paired-end reads closely related to PVs ranged between 15 and 92,000 in each sample and were de novo assembled into 41 distinct contigs using the SPAdes assembler (Table 1). RDP4 software was used to avoid the presence of chimeric genomes due to errors during assembly, and no chimeric sequences were observed. To simplify phylogenetic analyses, PV-related contigs were grouped into two different groups. The first group was composed of sequences that contained at least the complete L1 sequences, and the second group contained sequences with partial L1 longer than 350 bp. All L1 fragments shorter than 350 bp were not included in the phylogenetic analysis and are detailed in Additional file 1: Table S1.

**Table 1:** Overview of HTS results from the samples of this study

Identification sample	Total number of reads per sample	Number of BPV-related contigs	BPV related contigs length (nt)	Total number of BPV reads per contigs
3672RS16/BR	190,542	2	7215	509
			7202	92,705
3689RS16/BR	16,618	2	7139	6584
			783	2314
3690RS16/BR	264,016	1	2006	490
3880RS16/BR	232,396	1	1838	568
3891RS16/BR	233,268	3	5789	806
			1876	59
			1253	31
3895RS16/BR	87,096	5	741	13,062
			7288	406
			7333	2054
			7202	1469
			7201	23,782
3896RS16/BR	218,996	2	7619	292
			7233	2106
4147RS16/BR	252,508	2	7297	5766
			7288	39,794
4150RS16/BR	18,006	4	7699	36,477
			7276	70,481
			4131	3317
			2117	63
4151RS16/BR	19,142	1	3692	975
4182RS16/BR	202,168	1	4513	517
4826RS16/BR	222,824	3	7389	3767
			7333	41,028
			917	15
4827RS16/BR	212,292	9	7212	2343
			7241	11,596
			7634	3747
			7786	452
			7389	16,011
			7354	5184
			7271	6698
			7264	31,244
			7186	14,236
4828RS16/BR	223,378	2	7276	4701
			7205	3811
4833RS16/BR	219,088	1	7759	221
4834RS16/BR	21,761	1	7839	695
3654RS16/BR	268,184	ND	ND	ND
3670RS16/BR	351,53	ND	ND	ND
3682RS16/BR	254,736	ND	ND	ND
3686RS16/BR	290,652	ND	ND	ND
3694RS16/BR	1089	ND	ND	ND
4171RS16/BR	19,722	ND	ND	ND
4836RS16/BR	187,954	ND	ND	ND

*ND* not detected

### Complete known and new genomes

**Fig. 2:**
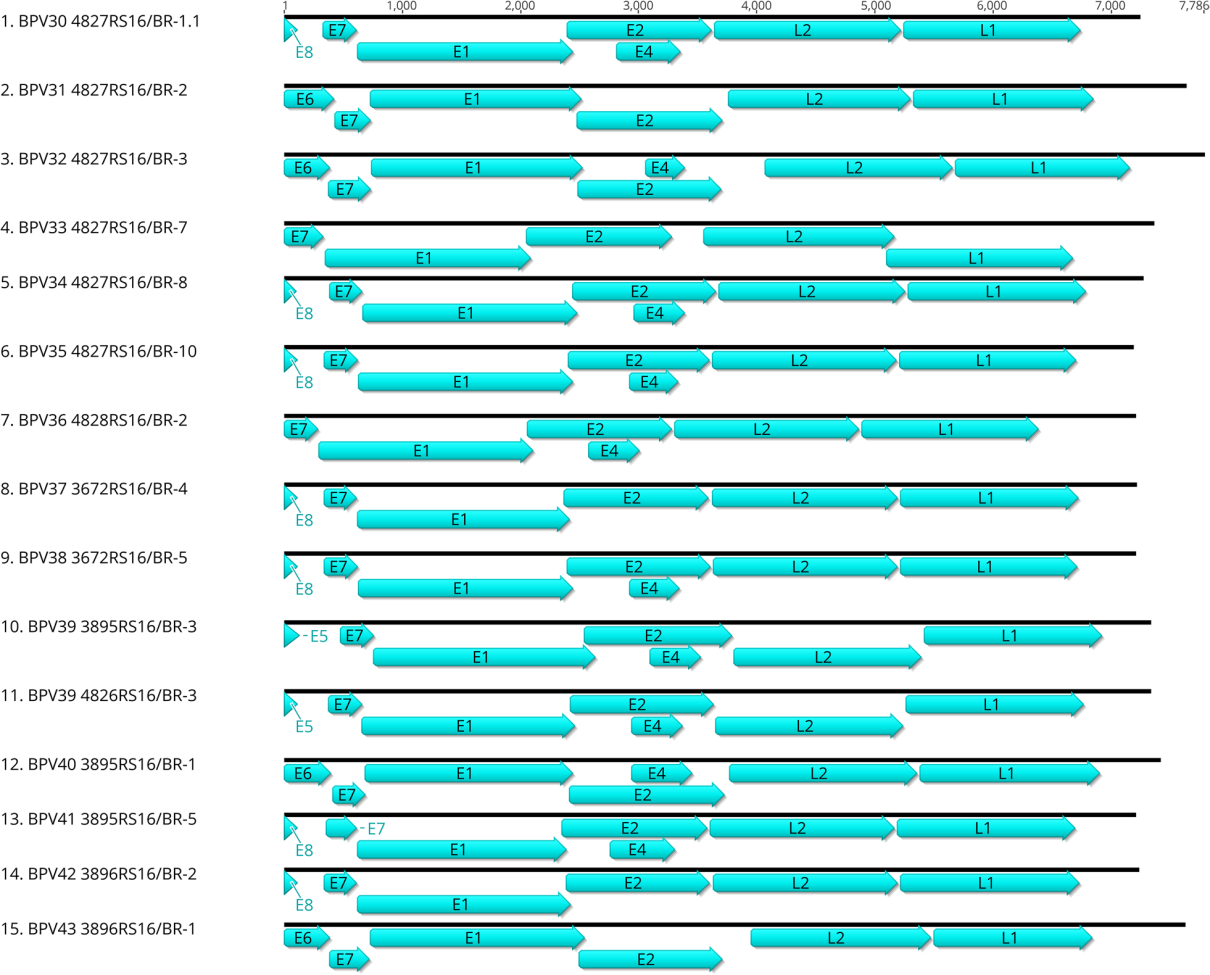
Genomic organization of the putative new BPV types found in this study. The first nucleotide in ORF5, ORF6, ORF7 or ORF8 was assigned number 1 in the sequences. The putative new BPV types contained five to eight ORFs encoding early (E1, E2, E4, E5, E6, E7 and E8) and late (L1 and L2) proteins

Twenty-six complete PV genomes were assembled in this study. In total, 11 genomes were from seven previously described BPV types and 15 genomes were from 14 new BPV types that are described herein (Additional file 2: Table S2). Eleven out of the 26 complete genomes detected in the present study from seven different samples were known BPVs (BPV3, 4, 6, 8, 9, 12 and 27). Seven complete genomes of the *Xipapillomavirus* genus were recovered from five distinct samples: BPV3 (two genomes, samples 4150RS16/BR and 4828RS16/BR), BPV4 (one genome, sample 4827RS16/BR), BPV6 (one genome, sample 4147RS16/BR), BPV9 (two genomes, samples 3895RS16/BR and 4147RS16/BR) and BPV12 (one genome, sample 3895RS16/BR), and the complete genome sequences were deposited in GenBank (accession numbers MW428431, MW428432, MW436424, MW436425, MW436426, MW436427, and MW436428, respectively). Two BPV8 (*Epsilonpapillomavirus* genus) and two BPV27 (unclassified) whole genomes were found in two distinct samples each (4834RS16/BR, 4150RS16/BR, 4826RS16/BR and 4827RS16/BR). The two complete BPV8 and the two complete BPV27 genomes were deposited in GenBank under accession numbers MW436430, MW436429, MW447310, and MW447311, respectively (Table 2). A total of 15 complete genomes from 14 new BPV types were detected and described in the present study (Fig. 2).

**Table 2:** GenBank accession numbers of complete and partial L1 sequences identified in the present study

Sequence identification	GenBank accession number	L1 sequence
BPV3 4150RS16/BR-2	MW428431	Complete
BPV3 4828RS16/BR-1	MW428432	Complete
BPV4 3891RS16/BR-3	MW393860	Complete
BPV4 4827RS16/BR-9	MW436424	Complete
BPV6 4147RS16/BR-3	MW436425	Complete
BPV8 4150RS16/BR-1	MW436429	Complete
BPV8 4834RS16/BR-2	MW436430	Complete
BPV9 3895RS16/BR-2	MW436426	Complete
BPV9 4147RS16/BR-4	MW436427	Complete
BPV11 4827RS16/BR-1	MW393861	Complete
BPV12 3895RS16/BR-4	MW436428	Complete
BPV27 4826RS16/BR-2	MW447310	Complete
BPV27 4827RS16/BR-6	MW447311	Complete
BPV29 4150RS16/BR-4	MW447312	Complete
BPV30 4827RS16/BR-1.1	MW390885	Complete
BPV31 4827RS16/BR-2	MW401529	Complete
BPV32 4827RS16/BR-3	MW401530	Complete
BPV33 4827RS16/BR-7	MW401531	Complete
BPV34 4827RS16/BR-8	MW404256	Complete
BPV35 4827RS16/BR-10	MW404257	Complete
BPV36 4828RS16/BR-2	MW404258	Complete
BPV37 3672RS16/BR-4	MW404259	Complete
BPV38 3672RS16/BR-5	MW404260	Complete
BPV39 3895RS16/BR-3	MW428425	Complete
BPV39 4826RS16/BR-3	MW428426	Complete
BPV39 3690RS16/BR-2	MW727481	Complete
BPV40 3895RS16/BR-1	MW428427	Complete
BPV41 3895RS16/BR-5	MW428428	Complete
BPV42 3896RS16/BR-2	MW428429	Complete
BPV43 3896RS16/BR-1	MW428430	Complete
BPV43 4182RS16/BR-1	MW543422	Complete
PNT 3880RS16/BR-6	MW543423	Complete
BPV7 4151RS16/BR-2	MW543418	Partial (> 350 bp)
BPV7 4151RS16/BR-6	MW543419	Partial (> 350 bp)
BPV25 4833RS16/BR-1	MW543420	Partial (> 350 bp)
PNT 3689RS16/BR-1	MW543421	Partial (> 350 bp)
PNT 3689RS16/BR-51	MW727480	Partial (> 350 bp)
PNT 3891RS16/BR-39	MW543426	Partial (> 350 bp)
PNT 4150RS16/BR-9	MW543427	Partial (> 350 bp)
PNT 3891RS16/BR-11	MW543424	Partial (> 350 bp)
PNT 4826RS16/BR-74	MW543425	Partial (> 350 bp)

**Fig. 3:**
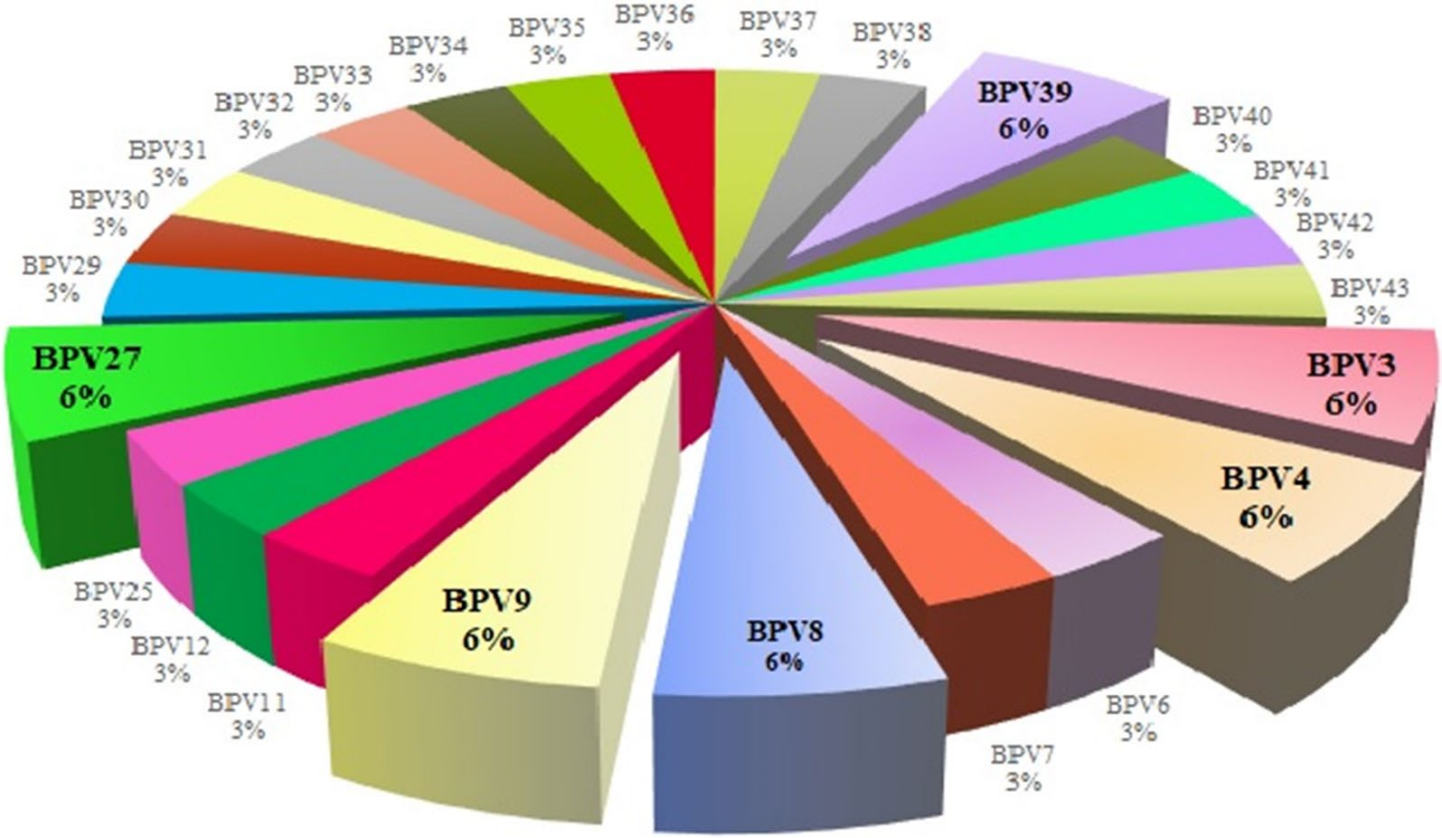
Frequency of different BPV types in the samples of this study. The graphic depicts the frequency of classical BPV types and new BPV types detected in the 16 samples with BPV-related contigs. BPV3, BPV4, BPV8, BPV9, BPV27 and the new BPV type named BPV39 were the most frequent types found in sequences greater than 350 bp

In the present study, the method enabled the detection of 11 classical BPVs as well as 14 new and five putative new BPV types. Furthermore, 56.3% of the samples (9/16) were coinfected by two or more PVs (Fig. 1), yielding two, three, five and nine distinct PV types found in four, three, one and one samples, respectively. The frequency of BPV types detected in the present study is detailed in Fig. 3, where BPV3, 4, 8, 9, 27 and the new type herein named BPV39 were the most frequent.

Herein, the putative new BPV types described were putatively named BPV30, BPV31, BPV32, BPV33, BPV34, BPV35, BPV36, BPV37, BPV38, BPV39 (two strains), BPV40, BPV41, BPV42 and BPV43, and the complete genomes were deposited under GenBank accession numbers MW390885, MW401529, MW401530, MW401531, MW404256, MW404257, MW404258, MW404259, MW404260, MW428425, MW428426, MW428427, MW428428, MW428429, and MW428430 (Table 2). One of the 14 putative new types, named BPV39, was composed of two different strains found in two distinct samples (3895RS16/BR and 4826RS16/BR). Ten of the new genomes (BPV30, 34, 35, 36, 37, 38, 39, 40, 41 and 42) were classified as the *Xipapillomavirus* genus according to phylogenetic analysis (nucleotide identity 71.1% to 86.1%). Additionally, the putative types BPV31, 32, 33 and 43 are probably new species and were classified into the *Dyoxipapillomavirus*, *Epsilonpapillomavirus*, unclassified and *Epsilonpapillomavirus* genera, respectively, after demonstrating low nucleotide identity with the closest PV sequences already described.

BPV31 was more related to BPV7 (NC_007612), a *Dyoxipapillomavirus* member, showing 63% nucleotide identity at the L1 gene. BPV32 and 43 were more related (nucleotide identity 66.9% and 70%) to *Epsilonpapillomavirus* genus members (GenBank accession numbers KU350625 and MG252779). BPV32 showed 66.9% L1 nucleotide identity with *Cervus elaphus papillomavirus* 1 (CePV1) (MN985322), and BPV43 showed 70% L1 nucleotide identity with BPV25 (MG252779). BPV33 presented 61.7% nucleotide identity with *Rusa timorensis papillomavirus* 2 (RtiPV2) (KT852571), which is still not classified in the PV genus.

The new PV genomes were predicted to contain five to seven ORFs that encoded three to five early genes (E1, E2, E4, E5, E6, E7, and E8) and two late genes (L1 and L2). The putative E6 proteins (present in the BPV31, 32, 40 and 43 genomes) contain a conserved zinc-binding domain (CXXC-X29-CXXC); however, they did not have a PDZ-binding motif (ETQL) in their C-terminus. All the E7 predicted proteins from the 14 new BPV types contained the conserved zinc-binding domain (CXXC-X29-CXXC). Curiously, the BPV30 E7 protein lacks the zinc-binding domain. The predicted E7 protein also has a retinoblastoma (pRb) protein-binding site (LxCxE) (BPV30, 34, 35, 36, 37, 38, 39, 40, 41 and 42); however, the BPV33 and BPV43 E7 proteins presented modified pRb protein-binding sites, which were slightly modified as LxExE/LxCxC/LxCxF and LxCxT, respectively. The novel E1 proteins were predicted to contain the conserved ATP-binding site (GPPDTGKS) of an ATP-dependent helicase; however, each one presented at least one mismatch. The leucine zipper domain (LX6LX6LX), which is present in some E2 proteins (BPV30, 34, 35, 36, 37, 38, 39, 40, 41 and 42), has at least one mismatch in these sequences. BPV32 and BPV33 E2 proteins do not have the leucine-zipper domain. The leucine-zipper domain was absent in the BPV31 and BPV43 E2 proteins but was present in the E1 protein. The late region encodes the viral capsid proteins L1 and L2, and the new sequences also present the already described high proportion of lysine and arginine (K and R) at the carboxy termini of both. The LCR regions also contain the predicted typical E1-binding sites (E1BS, ATTGTTN3AACAAT) as well as modified E1BS, typical E2-binding sites (E2BS, ACCN6GGT) and polyadenylation sites (polyA) (AATAAA) (Additional file 5: Table S5).

### Partial genomes

We also detected six partial PV genome sequences in six distinct samples that contained the complete L1 sequence. Three complete L1 sequences showed greater than 90% identity with reported BPV types (BPV4, 11 and 29) (GenBank accession numbers MW393860, MW393861, and MW447312, respectively), whereas two showed high identity with the new types herein described BPV39 and BPV43 (GenBank accession numbers MW727481 and MW543422, respectively) (Table 2). One complete L1 sequence (3880RS16/BR-6) displayed less than 90% nt identity with any PV sequence available in GenBank and was classified as a putative new BPV type (PNT) (GenBank accession number MW543423) (Additional file 3: Table S3; Table 2).

### Phylogenetic analysis of complete L1

**Fig. 4:**
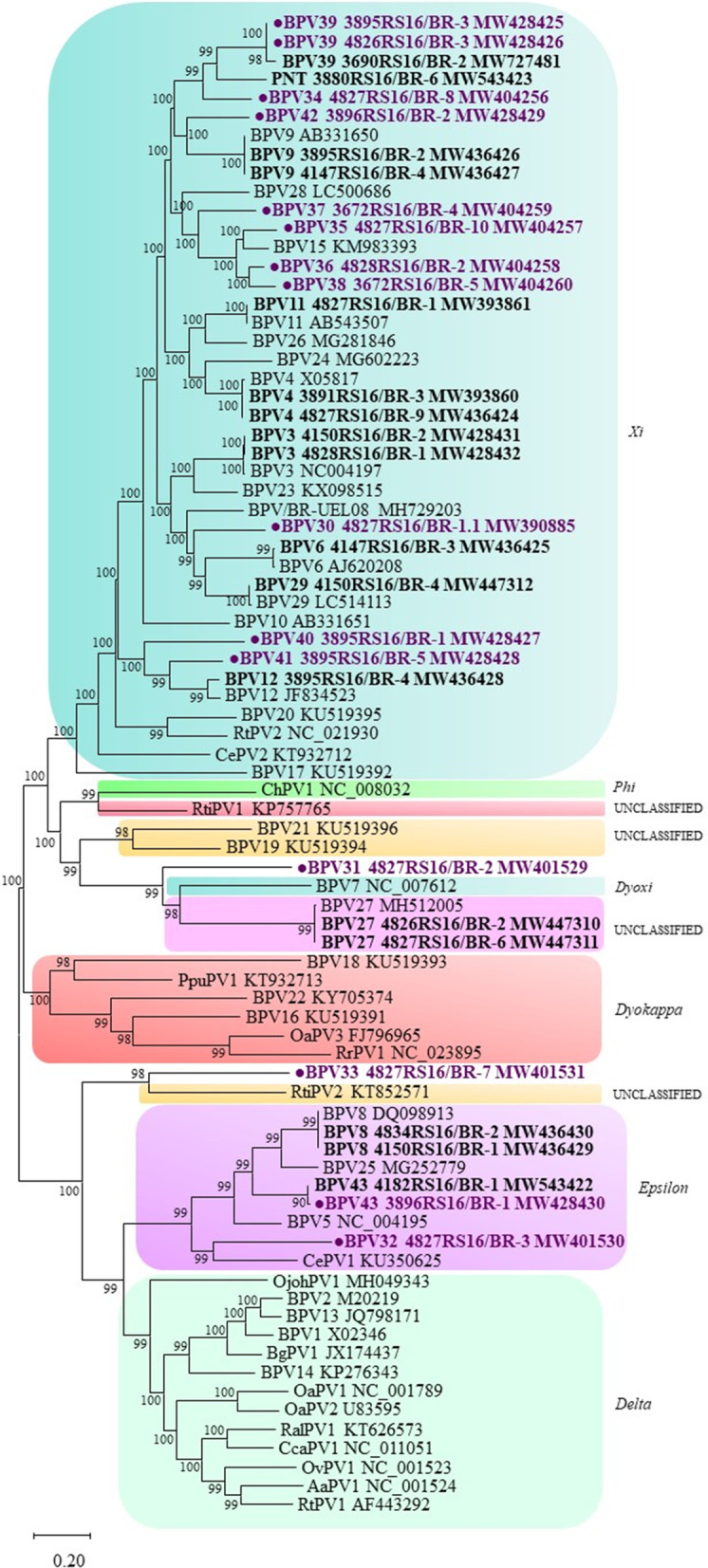
Molecular phylogenetic analysis of ruminant papillomavirus based on complete L1 gene nucleotide sequences. Complete L1 sequences were retrieved from GenBank and aligned with MAFFT, and phylogenetic analysis was performed using MEGA X software. The evolutionary history was inferred using the maximum likelihood method and general time reversible model. Numbers at internal nodes represent the bootstrap support values (percentages) determined for 1000 replications. The analysis involved 80 nucleotide sequences, and a total of 1383 positions were included in the final dataset. The sequences found in this study are represented in bold, and the putative new BPV types are represented by a purple circle. *PNT* putative new type

A phylogenetic tree was reconstructed using alignments based on the complete nucleotide sequence of the complete L1 gene using a set of ruminant PV-representative sequences [2] (Fig. 4). We observed that the novel BPV types most related to *Xipapillomavirus* members grouped within the *Xipapillomavirus* clade, which was supported by a 100% bootstrap value. Two putative new species found in this study (BPV32 and BPV43) grouped in the *Epsilonpapillomavirus* clade. The novel BPV31 constituted a distinct cluster presenting the same common ancestor of the *Dyoxipapillomavirus* genus and the unclassified genus that contains BPV27, which was supported by a bootstrap value of 99%. This new BPV type was most closely related to BPV7. Based on nucleotide sequence and phylogenetic tree analyses, this type probably represents a new species within the *Papillomaviridae* family. Furthermore, we observed that BPV33 clustered in a separate branch in the same terminal node of *Rusa timorensis papillomavirus* 2 (RtiPV2) (unclassified genus), which was supported by a bootstrap value of 98%. This finding suggests that this BPV type is likely a new species within the same unclassified genus.

### Analysis of partial L1

In the present study, we also detected 69 partial L1 sequences, nine of which were > 350 bp (Additional file 4: Table S4) and 60 were < 350 bp (Additional file 1: Table S1). In the analyses of L1 partial sequences greater than 350 bp long, we recovered two partial sequences of BPV7 from the same sample, both showing 99.6% nucleotide identity with the reference genome (NC_007612). Furthermore, another partial L1 sequence was also detected, showing 99.8% L1 nucleotide identity with the reference genome BPV25 (MG252779) isolate 14RS13/BR (Additional file 4: Table S4).

Our study identified four partial L1 sequences that displayed higher nucleotide identity with representatives of the *Xipapillomavirus* genus (BPV12, 17 and 15). The nucleotide identity of all these sequences ranged between 71.5% and 77%. Moreover, the same partial L1 gene recovered in two distinct samples (3891RS16/BR and 4826RS16/BR) was closely related to *Rusa timorensis papillomavirus* 2 (RtiPV2 KT852571) and showed 66.5% L1 sequence identity with the reference genome.

**Fig. 5:**
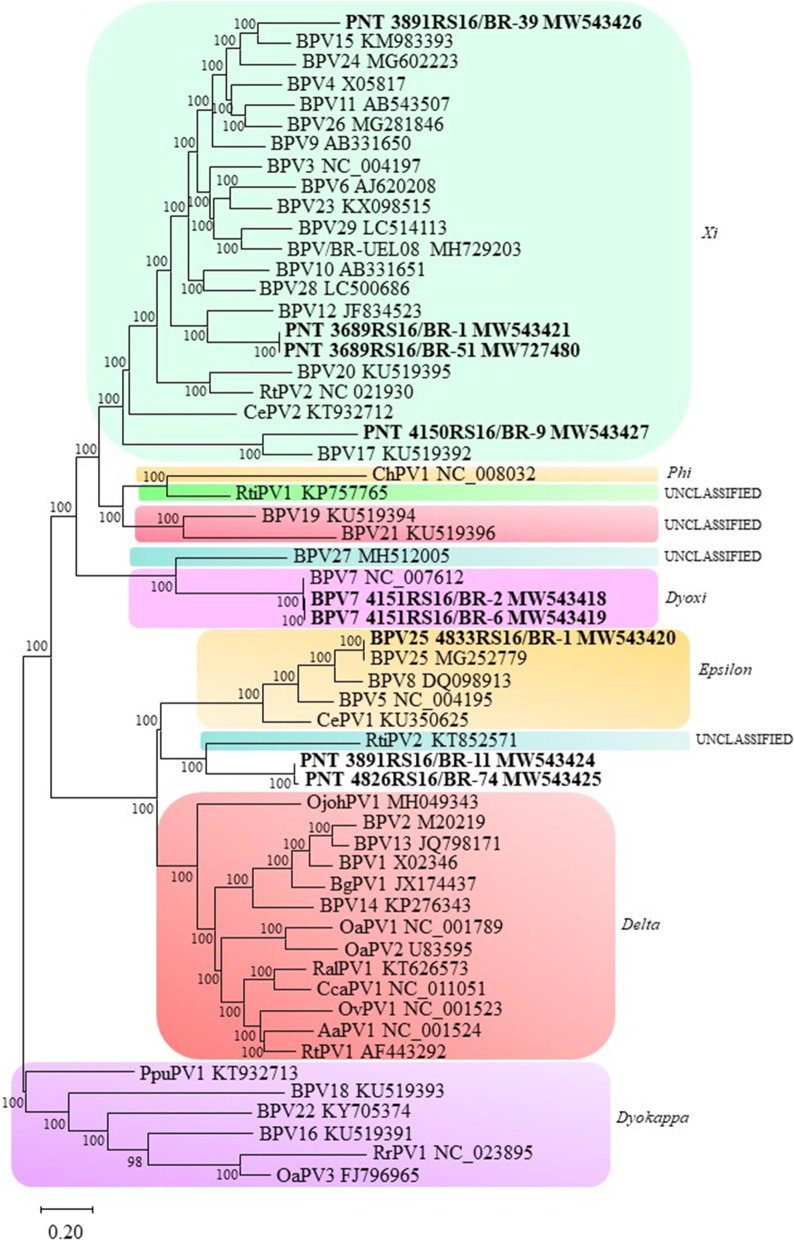
Molecular phylogenetic analysis of ruminant papillomavirus based on partial L1 gene nucleotide sequences. Complete L1 sequences were retrieved from GenBank and aligned with MAFFT, and phylogenetic analysis was performed using MEGA X software. The evolutionary history was inferred using the maximum likelihood method and general time reversible model. Numbers at internal nodes represent the bootstrap support values (percentages) determined for 1000 replications. The analysis involved 57 nucleotide sequences, and there were a total of 445 positions in the final dataset. The sequences found in this study are represented in bold. *PNT* putative new type

The phylogenetic analysis was performed with the partial L1 sequences using a set of ruminant PV sequences (Fig. 5). The partial L1 sequences found in this study are more closely related to *Xipapillomavirus* members clustered into this clade, and this notion is supported by a 100% bootstrap value. We observed that the sequences closely related to RtiPV2 (unclassified genus) clustered in a separated branch and likely represent a putative new BPV type within the same unclassified genus.

## Discussion

The present study investigated BPV coinfections and genetic diversity in teat papillomatous lesions using an RCA-HTS approach. Studies related to an increase in animal neoplasms [26] and its association with PV are already well recognized [27]. In cattle, PV is associated with benign and malignant tumors, and teat papillomatosis can predispose dairy cows to secondary infections and milk discard [2, 28]. Nevertheless, there is a lack of research on animal PV [9, 10], contrasting with the large number of studies involving HPV [14, 15]. Moreover, the works that evaluated coinfections in cattle only used a limited number of animals [21, 23] in contrast with the 23 used in the present study. Additionally, it is well known the HPV coinfections and HPV combination may act as an increasing factor for carcinogenesis [14, 16, 18].

The identification of coinfections in this study corroborates some studies in cattle that have demonstrated that the occurrence of BPV coinfections is a common event [20, 29], as previously observed with HPV [14, 15]. Another study using the RCA-HTS approach enabled the detection of seven dissimilar PV genomes in a single epidermal papillomatosis lesion that belongs to *Delta*, *Xi*, and *Dyokappapapillomavirus* genera and an unclassified genus [23]. Herein, we observed the same high frequency of coinfections, and one sample contained nine PVs from the *Xi*- and *Epsilonpapillomavirus* genera and two unclassified genera. Although BPV coinfection was detected in 21.4% of the samples analyzed in an outbreak of teat papillomatosis and double mixed infections were reported in teat and healthy skin samples from cattle [30], these are low frequencies of coinfections compared to our data (56.3% of the samples coinfected). However, more studies are necessary to understand the real diversity of BPV as well as the importance of mixed infections in papillomatous lesions. Moreover, the data presented in the present study reinforced by previous studies [20, 29–31] suggest that coinfections must be considered in papillomatosis and neoplastic transformations. Nevertheless, studies to assay the frequency of PV coinfection in cattle remain limited.

Corroborating the low number of studies about PV diversity in cattle [20, 23, 31, 32], our study revealed a high genetic PV diversity in teat papillomas, where 15 new complete BPV genomes were identified as 14 putative new types. Additionally, we detected 11 classical BPV types belonging to the *Xi*, *Epsilon* and *Dyoxipapillomavirus* genera and five putative new BPV types.

Within these classical BPV types, we found two BPV27 complete genomes. To the best of our knowledge, this is the first report of BPV27 in Brazil. The BPV27 genomes found in this study showed high nucleotide identity (99.7%) in the L1 gene sequence with each other compared to the strain detected in a dairy cow in China [33], suggesting that this BPV type may be globally widespread and underdetected.

Classically, BPV types are believed to have a tropism for specific anatomic sites. Previous studies demonstrated that BPV6, 7, 9 and 10 were associated with the etiology of teat papillomas [10, 31, 34]. However, the identification of several BPV types in teat papillomas evaluated herein showed the high diversity of PV types involved with this disease.

Until 2016, only 15 BPV types were fully sequenced and characterized given that conventional PCR using degenerate primer pairs selectively amplifies a virus population with high affinity to these primers and fails to detect phylogenetically distant PVs [23]. However, since the application of the RCA-HTS approach, a significant number of new BPV types have been described as well as coinfections in the same papilloma lesion [23, 35, 36]. This strategy applied herein identified viruses that included known papillomaviruses as well as putative and novel PV types that differ from those previously reported. The RCA method has been used for the efficient amplification of circular DNA viral genomes without the need for specific primers [37], facilitating the rapid increase in novel PV type knowledge. This technique can lead to the identification of a large number of new PV types, which remain underestimated using the conventional approach [23, 37]. RCA-HTS has no PV selective amplification. This approach can amplify not only PVs but also several viruses with circular double-stranded DNA with a more general use [37, 38]. Conventional PCR using the degenerate primer pair FAP59/FAP64 amplifies a relatively conserved fragment of the L1 gene from all known PV types [25]. This approach can fail to detect new PV genomes present in papilloma lesions due to lower homology of the base pairing in the 3’ region of both primer binding sites [23] (Additional file 6: Fig. S1).

The detection of two putative new BPV types (BPV32 and BPV33) closely related to *Cervidae papillomavirus* may suggest that these putative new BPV types could have originated from a common ancestor. These findings support the hypothesis of a primitive papillomavirus, which becomes more species specific by continued replication in their respective host. This coevolution may justify the similarities between these viruses [39, 40].

Generally, the PV genomes encode at least one of the three viral oncogenes (E5, E6 and E7), which are involved in viral proliferation and in the host-cell transformation process that leads to the proliferation of cancer cells [2, 39]. Deltapapillomaviruses are widely detected in bovines, contain a complete set of oncogenes, are well known for their involvement in malignant cell transformation and are classified as high-risk BPVs [2]. However, no *Delta*-PV was detected in the present study. Deltapapillomaviruses are commonly involved in fibropapillomas and cutaneous papillomatosis [2]. These papillomaviruses are typically identified by conventional PCR using the degenerate FAP primer pair [41, 42] due to the higher homology of oligonucleotide annealing in the 3´ region of primer binding sites (Additional file 6: Fig. S1).

The genomic organization of the new BPV types revealed the presence of the three oncogenes (E5, E6 and E7) [2]; however, none presented the complete oncogene set (E5 and E7 or E6 and E7 or only E7) (Fig. 2), suggesting that these new BPV genomes may not be highly oncogenic. The majority of the new genomes encoded only the E7 ORF (*Xipapillomavirus*: BPV30, 34, 35, 36, 37, 38, 41 and 42; unclassified: BPV33), whereas others encompassed both E6 and E7 (*Xipapillomavirus*: BPV40; *Epsilonpapillomavirus*: BPV32 and BPV43; *Dyoxipapillomavirus*: BPV31) or E5 and E7 oncogenes (*Xipapillomavirus*: BPV39). The BPV oncoprotein E5 is the major transforming protein, showing malignancy activity and leading to cell transformation and tumorigenesis; however, it is not present in all BPV genera [2, 43]. Previous studies showed that the E5 protein is present in *Deltapapillomavirus*, *Epsilonpapillomavirus* and in a variety of *Xipapillomavirus* genomes [2], which were detected in the present study (*Xipapillomavirus*: BPV30, 34, 35, 36, 37, 38, 39, 40, 41 and 42; *Epsilonpapillomavirus*: BPV32 and BPV43). PV animal tumors are considered important models to understand the human oncogenic process, allowing the identification of mechanisms involved in carcinogenesis [26]. Therefore, it is important to study animal PV by performing genetic characterizations of complete genomes to understand the PV genetics and their potential pathogenicity, as well as the genetic diversity of this wide group of oncogenic viruses.

The PV oncoproteins E6 and E7 bind to host proteins and modulate cell differentiation. In the E7 ORF, the LxCxE motif has been associated with host cell transformation. Twelve of the new *Xipapillomavirus* genomes harbored this conserved motif, similar to other *Xipapillomavirus* species [2]. However, some LxCxE motifs exhibited slightly modified amino acid sequences in the BPV33 and BPV43 E7 ORFs. In BPV33, the cysteine residue was replaced by glutamic acid (C → E), and the glutamic acid residue was replaced by cysteine and phenylalanine (E → C; E → F). Moreover, in BPV43, glutamic acid was replaced by a threonine residue (E → T). Because all the amino acids have different hydrophobic side groups, this substitution could pose problems for the functionality of the pRb-binding site domain. Another conserved motif observed in the PV E6 ORF is the PDZ-binding motif (ETQL), which is necessary for the induction of epithelial hyperplasia [11]. Similar to other Ruminantia PV E6 ORFs [2], all the new E6 proteins found herein lack this domain in their carboxy-terminal amino acids.

## Conclusions

In the present study, we report 26 BPV complete genome sequences detected in 23 teat papillomatous lesions from dairy cattle located in southern Brazil, including 14 new BPV types herein named BPV30 to BPV43. Additionally, several complete and partial genomes of known and new BPV types were found coinfecting the same papilloma lesion and confirmed that BPV coinfection is a frequent event that may putatively act synergistically in the lesion. The data generated in this study suggest the existence of numerous other BPV types that could have been under detected by conventional approaches and added 14 new BPV-characterized genomes to the 29 previously known genomes. Furthermore, future studies should be conducted to better understand BPV diversity and biology and their role in papilloma and neoplastic lesions.

## Methods

### Samples

The 23 teat wart samples used in the present study were collected in abattoirs from milking cows that were discarded in 2016 in two cities located in Rio Grande do Sul, southern Brazil: Passo Fundo (10 samples) and Farroupilha (13 samples). The collected animals were selected due the presence of papilloma-like lesions in udder. The samples were previously diagnosed with epidermal papillomatosis [9].

### DNA isolation

Papilloma samples were ground with a mortar and pestle in 1 mL of phosphate buffered saline (PBS) pH 7.4 and centrifuged at 720×*g* for 10 min, and the supernatant was stored at − 80 °C for molecular analysis. Total DNA was isolated in 100 µL of sample using a phenol–chloroform-based protocol [44]. The quantity and quality of the DNA were assessed by spectrophotometry using a NanoDrop™ (Thermo Fisher Scientific).

### Rolling-circle amplification (RCA) and high-throughput sequencing (HTS)

Viral DNA was enriched by randomly primed RCA [23, 37, 41] using a commercial kit (TempliPhi™100 Amplification Kit, GE Healthcare) to enrich circular DNA according to the manufacturer’s instructions. The amplicons were electrophoresed in a 0.5% agarose gel and visualized on a UV light source. The RCA products were purified using a commercial kit (Purelink Quick PCR Purification Kit, Thermo Fisher Scientific®), and the quality and quantity of the DNA were assessed by spectrophotometry and fluorometry using NanoDrop™ (Thermo Fisher Scientific®) and Qubit™ (Thermo Fisher Scientific®), respectively. DNA fragment libraries were prepared with 50 ng of purified DNA using a Nextera XT DNA sample preparation kit (Illumina, USA) and sequenced using an Illumina MiSeq System (Illumina, USA) using an Illumina v2 reagent kit (2 × 150 paired end reads).

### Bioinformatic analysis

The quality of the read libraries from the DNA sequencing was evaluated using FastQC. Furthermore, the reads were 3' trimmed based on a Phred quality score cutoff of 20 using Geneious software (version 9.0.5) [45]. Subsequently, read libraries were assembled into contigs with SPAdes (version 3.9.0) [46] followed by confirmation by mapping the libraries back to the assembled contigs using Geneious software. Contigs related to PV were evaluated with RDP4 software [47] to avoid the presence of chimeric genomes and then compared to sequences in GenBank nucleotide and protein databases using BLASTn/BLASTx. For alignments, open reading frame (ORF) prediction and genome annotation were performed using Geneious software.

### Sequence analysis

L1 ORF complete sequences of ruminant papillomaviruses were aligned with MAFFT [48]. MEGA X was used for phylogeny inference. A tree was generated using the best selection model defined with MEGA X as well as the maximum likelihood method. General time reversible with gamma distribution and invariant sites (GTR + G + I) was performed with 1,000 nonparametric bootstrap analyses [49, 50]. The PV classification is based on the nucleotide sequence identity of the L1 gene. A new PV type is defined when the entire genome is sequenced, and the entire L1 gene differs by greater than 10% compared with any PV type. To be considered a new PV species, the L1 gene would have less than 29% nucleotide identity with all the PV types already identified. Furthermore, new PV genera were defined as those displaying less than 60% nucleotide identity of the L1 gene [1, 3].

### Data availability

Sequence data that support the findings of this study have been deposited in GenBank with the accession numbers MW428431 (BPV3), MW428432 (BPV3), MW436424 (BPV4), MW436425 (BPV6), MW436426 (BPV9), MW436427 (BPV9), MW436428 (BPV12), MW436430 (BPV8), MW436429 (BPV8), MW447310 (BPV27), MW447311 (BPV27), MW390885 (BPV30), MW401529 (BPV31), MW401530 (BPV32), MW401531 (BPV33), MW404256 (BPV34), MW404257 (BPV35), MW404258 (BPV36), MW404259 (BPV37), MW404260 (BPV38), MW428425 (BPV39), MW428426 (BPV39), MW428427 (BPV40), MW428428 (BPV41), MW428429 (BPV42), MW428430 (BPV43), MW393860 (BPV4), MW393861 (BPV11), MW447312 (BPV29), MW543422 (BPV43), MW727481 (BPV39), MW543423 (PNT 3880RS16/BR-6), MW543418 (BPV7), MW543419 (BPV7), MW543420 (BPV25), MW543421 (PNT 3689RS16/BR-1), MW727480 (PNT 3689RS16/BR-51), MW543424 (PNT 3891RS16/BR-11), MW543426 (PNT 3891RS16/BR-39), MW543427 (PNT 4150RS16/BR-9), and MW543425 (PNT 4826RS16/BR-74). The authors declare that the data supporting the findings of this study are available within the paper and its supplementary information files.

## Supplementary Information

**Additional file 1.** Pairwise identity between partial L1 sequences (< 350 bp) from BPV samples from this study compared with the more related sequence available in GenBank.

**Additional file 2.** Description of nucleotide identity between complete genome sequences found in this study compared with sequences available in GenBank. Genomes recovered from the same sample are sequentially numbered after the sample name.

**Additional file 3.** Percentage of nucleotide identity between L1 gene complete sequences from this study compared with sequences available in GenBank. L1 complete sequences recovered from the same sample are sequentially numbered after the sample name.

**Additional file 4.** Nucleotide identity between partial L1 gene sequences (> 350 bp) from this study compared with sequences available in GenBank. Sequences recovered from a single sample are sequentially numbered after the sample name.

**Additional file 5.** Conserved motifs of PV genomes, including nt positions of E2 and E1-binding site motifs, ATP binding site of the ATP-dependent helicase, leucine-zipper domain of E2 ORF, LxCxE motif of E7 ORFs, zinc-binding domains of E6 and E7 ORFs and polyadenylation sites.

**Additional file 6.** FAP primer pair annealing sites in all the new PV L1 sequences. Alignment of the BPV L1complete sequences demonstrating the annealing efficiency between the sequences of this study and classical BPVs (Delta, Xi, Epsilon, Dyoxi and Dyokappapapillomavirus genera). The color gradation shows the degree of nucleotide identity, where black represents 100% identity between the nucleotide sequences. In the upper part, the graph indicates a summary of the most frequent nucleotides. Nucleotide alignment was generated using MUSCLEaligner with default settings in Geneious software v. 9.0.5.
