# Supplementary material for: The genetic diversity of “papillomavirome” in bovine teat papilloma lesions
Source: Anim Microbiome. 2021 Jul 28;3:51. doi: 10.1186/s42523-021-00114-3 (PMC8317299; doi:10.1186/s42523-021-00114-3)
Supplement: Supplementary file 1 — Additional file 1. Pairwise identity between partial L1 sequences (< 350 bp) from BPV samples from this study compared with the more related sequence available in GenBank. [file 42523_2021_114_MOESM1_ESM.docx]

**Table S1.** Pairwise identity between partial L1 sequences (< 350 bp) from BPV samples from this study compared with the more related sequence available in GenBank.

| **Sample** | **Contig** | **Lenght (nt)** | **Lenght (aa)** | **Best BLASTn hit**  **(BPV type/strain/GenBank accession number)** | **L1 nucleotide identity (%)** |
| --- | --- | --- | --- | --- | --- |
| 3670RS16/BR | 7 | 385 | 128 | BPV8/ strain BR/02AC12/ MH237829.1 | 100 |
| 3670RS16/BR | 1309 | 286 | 95 | BPV8/ strain BR/02AC12/ MH237829.1 | 99.18 |
| 3670RS16/BR | 2732 | 215 | 71 | BPV8/ strain 8-EB/ DQ098917.1 | 100 |
| 3670RS16/BR | 3420 | 50 | 16 | BPV8/ strain BR/02AC12/ MH237829.1 | 100 |
| 3670RS16/BR | 4992 | 167 | 55 | BPV8/ strain 8-EB/ DQ098917.1 | 100 |
| 3670RS16/BR | 6280 | 61 | 20 | BPV8/ strain BR/02AC12/ MH237829.1 | 100 |
| 3670RS16/BR | 8106 | 77 | 25 | BPV8/ strain BR/02AC12/ MH237829.1 | 100 |
| 3670RS16/BR | 8763 | 86 | 28 | BPV8/ strain BR/02AC12/ MH237829.1 | 100 |
| 3670RS16/BR | 8960 | 97 | 32 | BPV8/ strain BR/02AC12/ MH237829.1 | 100 |
| 3670RS16/BR | 9546 | 70 | 23 | BPV8/ strain BR/02AC12/ MH237829.1 | 100 |
| 3670RS16/BR | 10009 | 81 | 27 | BPV8/ strain BR/02AC12/ MH237829.1 | 100 |
| 3670RS16/BR | 10293 | 96 | 32 | BPV8/ strain BR/02AC12/ MH237829.1 | 100 |
| 3670RS16/BR | 11926 | 94 | 31 | BPV8/ strain 8-EB/ DQ098917.1 | 100 |
| 3670RS16/BR | 14616 | 61 | 20 | BPV8/ strain 8-EB/ DQ098917.1 | 100 |
| 3670RS16/BR | 15453 | 84 | 28 | BPV8/ strain BR/02AC12/ MH237829.1 | 100 |
| 3670RS16/BR | 18687 | 71 | 23 | BPV8/ strain BR/02AC12/ MH237829.1 | 100 |
| 3670RS16/BR | 20631 | 65 | 21 | BPV8/ strain 8-EB/ DQ098917.1 | 100 |
| 3670RS16/BR | 22076 | 29 | 9 | BPV8/ strain BR/02AC12/ MH237829.1 | 100 |
| 3672RS16/BR | 1112 | 412 | 137 | BPV3/ AF486184.1 | 99.27 |
| 3672RS16/BR | 1212 | 330 | 109 | BPV29/ strain B191016/ LC514113.1 | 81.31 |
| 3672RS16/BR | 2328 | 75 | 24 | BPV3/ AF486184.1 | 100 |
| 3682RS16/BR | 620 | 126 | 41 | BPV8/ strain 8-EB/ DQ098917.1 | 100 |
| 3682RS16/BR | 4084 | 84 | 28 | BPV8/ strain BR/02AC12/ MH237829.1 | 100 |
| 3682RS16/BR | 4307 | 133 | 44 | BPV8/ strain BR/02AC12/ MH237829.1 | 100 |
| 3682RS16/BR | 11385 | 137 | 45 | BPV8/ strain BR/02AC12/ MH237829.1 | 100 |
| 3686RS16/BR | 2 | 138 | 45 | BPV11/ AB543507.1 | 85.61 |
| 3686RS16/BR | 967 | 212 | 70 | BPV/ strain BPV/CHI-SW2/ MH729202.1 | 98.71 |
| 3686RS16/BR | 7732 | 161 | 53 | BPV/ strain BPV/CHI-SW2/ MH729202.1 | 99.16 |
| 3686RS16/BR | 10032 | 135 | 45 | BPV/ strain BPV/CHI-SW2/ MH729202.1 | 100 |
| 3690RS16/BR | 17695 | 82 | 27 | Bovine papillomavirus/ strain BPV/BR-UEL6/ KP892554.1 | 91.78 |
| 3694RS16/BR | 1 | 279 | 92 | BPV25/ strain 14RS13/BR/ MG252779.1 | 89.29 |
| 3694RS16/BR | 171 | 153 | 50 | BPV25/ strain 14RS13/BR/ MG252779.1 | 89.55 |
| 3694RS16/BR | 862 | 144 | 47 | BPV25/ strain 14RS13/BR/ MG252779.1 | 91.67 |
| 3694RS16/BR | 1204 | 103 | 34 | BPV25/ strain 14RS13/BR/ MG252779.1 | 89.32 |
| 3694RS16/BR | 1478 | 103 | 34 | BPV25/ strain 14RS13/BR/ MG252779.1 | 89.32 |
| 3694RS16/BR | 5026 | 132 | 43 | BPV25/ strain 14RS13/BR/ MG252779.1 | 91.67 |
| 3880RS16/BR | 672 | 373 | 124 | BPV12 / strain PR000002/ JF834524.1 | 83.56 |
| 3896RS16/BR | 8231 | 46 | 15 | Bovine papillomavirus/ strain BPV/BR-UEL5/ EU293541.1 | 100 |
| 4147RS16/BR | 243 | 377 | 125 | BPV15/ strain Aks-02/ KM983393.1 | 79.08 |
| 4150RS16/BR | 118 | 286 | 95 | BPV12 / strain PR000002/ JF834524.1 | 75.46 |
| 4150RS16/BR | 957 | 242 | 80 | Bovine papillomavirus/ strain BPV/BR-UEL5/ EU293541.1 | 99.17 |
| 4151RS16/BR | 91 | 197 | 65 | BPV7/ DQ217793.1 | 98.98 |
| 4151RS16/BR | 1197 | 108 | 35 | BPV7/ strain IT-221/ KM096429.1 | 100 |
| 4151RS16/BR | 3192 | 197 | 65 | BPV7/ DQ217793.1 | 98.98 |
| 4151RS16/BR | 3369 | 266 | 88 | BPV7/ DQ217793.1 | 98.98 |
| 4151RS16/BR | 3519 | 106 | 35 | BPV7/ strain IT-221/ KM096429.1 | 99.06 |
| 4151RS16/BR | 4836 | 159 | 53 | BPV7/ strain IT-221/ KM096429.1 | 99.06 |
| 4151RS16/BR | 9571 | 145 | 48 | BPV7/ strain IT-221/ KM096429.1 | 100 |
| 4151RS16/BR | 18264 | 65 | 21 | BPV7/ strain IT-221/ KM096429.1 | 100 |
| 4182RS16/BR | 546 | 192 | 63 | Bovine papillomavirus/ strain BPV/BR-UEL5/ EU293541.1 | 93.55 |
| 4182RS16/BR | 2885 | 59 | 19 | BPV8/ strain BR/02AC12/ MH237829.1 | 88 |
| 4182RS16/BR | 3157 | 152 | 50 | Bovine papillomavirus/ strain BPV/BR-UEL5/ EU293541.1 | 94.32 |
| 4182RS16/BR | 5720 | 59 | 19 | BPV5/ strain BR/02AC12/ MH220402.1 | 88.10 |
| 4827RS16/BR | 2759 | 332 | 110 | BPV12 / strain PR000002/ JF834524.1 | 78.02 |
| 4833RS16/BR | 472 | 338 | 112 | BPV15/ strain Aks-02/ KM983393.1 | 83.58 |
| 4833RS16/BR | 1610 | 514 | 171 | BPV15/ strain Aks-02/ KM983393.1 | 81.46 |
| 4834RS16/BR | 791 | 108 | 36 | BPV8/ strain BR/02AC12/ MH237829.1 | 100 |
| 4834RS16/BR | 2858 | 242 | 80 | BPV8/ strain BR/02AC12/ MH237829.1 | 100 |
| 4834RS16/BR | 6355 | 110 | 36 | BPV8/ strain BR/02AC12/ MH237829.1 | 100 |
| 4834RS16/BR | 6964 | 86 | 28 | BPV8/ strain BR/02AC12/ MH237829.1 | 100 |
