# Supplementary material for: The genetic diversity of “papillomavirome” in bovine teat papilloma lesions
Source: Anim Microbiome. 2021 Jul 28;3:51. doi: 10.1186/s42523-021-00114-3 (PMC8317299; doi:10.1186/s42523-021-00114-3)
Supplement: Supplementary file 2 — Additional file 2. Description of nucleotide identity between complete genome sequences found in this study compared with sequences available in GenBank. Genomes recovered from the same sample are sequentially numbered after the sample name. [file 42523_2021_114_MOESM2_ESM.docx]

**Table S2.** Description of nucleotide identity between complete genome sequences found in this study compared with sequences available in GenBank. Genomes recovered from the same sample are sequentially numbered after the sample name.

| **BPV type/putative new BPV type** | **Sequence identification** | **Sample** | **Best BLASTn hit/GenBank accession number** | **L1 nucleotide identity (%)** |
| --- | --- | --- | --- | --- |
| BPV3 | 4150RS16/BR-2 | 4150RS16/BR | BPV3/ AF486184.1 | 98.83 |
| BPV3 | 4828RS16/BR-1 | 4828RS16/BR | BPV3/ AF486184.1 | 98.96 |
| BPV4 | 4827RS16/BR-9 | 4827RS16/BR | BPV4/ X05817.1 | 98.69 |
| BPV6 | 4147RS16/BR-3 | 4147RS16/BR | BPV6/ AJ620208.1 | 99.8 |
| BPV8 | 4150RS16/BR-1 | 4150RS16/BR | BPV8 strain 8-EB/ DQ098917.1 | 100 |
| BPV8 | 4834RS16/BR-2 | 4834RS16/BR | BPV8 strain 8-EB/ DQ098917.1 | 99.80 |
| BPV9 | 3895RS16/BR-2 | 3895RS16/BR | BPV9/ AB331650.1 | 99.56 |
| BPV9 | 4147RS16/BR-4 | 4147RS16/BR | BPV9/ AB331650.1 | 99.5 |
| BPV12 | 3895RS16/BR-4 | 3895RS16/BR | BPV12 strain PR000002/ JF834524.1 | 93.36 |
| BPV27 | 4826RS16/BR-2 | 4826RS16/BR | BPV27 strain ujs-21015/ MH512005.1 | 99.74 |
| BPV27 | 4827RS16/BR-6 | 4827RS16/BR | BPV27 strain ujs-21015/ MH512005.1 | 99.74 |
| BPV30 | 4827RS16/BR-1.1 | 4827RS16/BR | BPV29 strain B191016/ LC514113.1 | 77.31 |
| BPV31 | 4827RS16/BR-2 | 4827RS16/BR | BPV7/ DQ217793.1 | 65 |
| BPV32 | 4827RS16/BR-3 | 4827RS16/BR | BPV5 strain BR/02AC12/ MH220402.1 | 68.19 |
| BPV33 | 4827RS16/BR-7 | 4827RS16/BR | RtiPV2 strain IZW 39/08/ NC_040785.1 | 66.2 |
| BPV34 | 4827RS16/BR-8 | 4827RS16/BR | BPV9/ AB331650.1 | 76.52 |
| BPV35 | 4827RS16/BR-10 | 4827RS16/BR | BPV15 strain Aks-02/ KM983393.1 | 85.99 |
| BPV36 | 4828RS16/BR-2 | 4828RS16/BR | BPV15 strain Aks-02/ KM983393.1 | 86.52 |
| BPV37 | 3672RS16/BR-4 | 3672RS16/BR | BPV15 strain Aks-02/ KM983393.1 | 77.14 |
| BPV38 | 3672RS16/BR-5 | 3672RS16/BR | BPV15 strain Aks-02/ KM983393.1 | 84.46 |
| BPV39 | 3895RS16/BR-3 | 3895RS16/BR | BPV11/ AB543507.1 | 74.84 |
| BPV39 | 4826RS16/BR-3 | 4826RS16/BR | BPV11/ AB543507.1 | 74.84 |
| BPV40 | 3895RS16/BR-1 | 3895RS16/BR | BPV12 strain PR000002/ JF834524.1 | 71.18 |
| BPV41 | 3895RS16/BR-5 | 3895RS16/BR | BPV12 strain PR000002/ JF834524.1 | 78.61 |
| BPV42 | 3896RS16/BR-2 | 3896RS16/BR | BPV15 strain Aks-02/ KM983393.1 | 75.54 |
| BPV43 | 3896RS16/BR-1 | 3896RS16/BR | BPV25/ strain 14RS13/BR/ MG252779.1 | 77.13 |
