# Supplementary material for: The genetic diversity of “papillomavirome” in bovine teat papilloma lesions
Source: Anim Microbiome. 2021 Jul 28;3:51. doi: 10.1186/s42523-021-00114-3 (PMC8317299; doi:10.1186/s42523-021-00114-3)
Supplement: Supplementary file 3 — Additional file 3. Percentage of nucleotide identity between L1 gene complete sequences from this study compared with sequences available in GenBank. L1 complete sequences recovered from the same sample are sequentially numbered after the sample name. [file 42523_2021_114_MOESM3_ESM.docx]

**Table S3.** Percentage of nucleotide identity between L1 gene complete sequences from this study compared with sequences available in GenBank. L1 complete sequences recovered from the same sample are sequentially numbered after the sample name.

| **BPV type/putative new BPV type** | **Sequence identification** | **Sample** | **Best BLASTn hit/GenBank accession number** | **L1 nucleotide identity (%)** |
| --- | --- | --- | --- | --- |
| BPV4 | 3891RS16/BR-3 | 3891RS16/BR | BPV4/ X05817.1 | 98.69 |
| BPV29 | 4150RS16/BR-4 | 4150RS16/BR | BPV29 strain B191016/ LC514113.1 | 99.34 |
| BPV11 | 4827RS16/BR-1 | 4827RS16/BR | BPV11/ AB543507.1 | 99.67 |
| putative new BPV43 | 4182RS16/BR-1 | 4182RS16/BR | BPV25/ strain 14RS13/BR/ MG252779.1 | 77.61 |
| putative new BPV type | 3880RS16/BR-6 | 3880RS16/BR | BPV11/ AB543507.1 | 75.62 |
| putative new BPV39 | 3690RS16/BR-2 | 3690RS16/BR | BPV11/ AB543507.1 | 74.77 |
