# Supplementary material for: The genetic diversity of “papillomavirome” in bovine teat papilloma lesions
Source: Anim Microbiome. 2021 Jul 28;3:51. doi: 10.1186/s42523-021-00114-3 (PMC8317299; doi:10.1186/s42523-021-00114-3)
Supplement: Supplementary file 4 — Additional file 4. Nucleotide identity between partial L1 gene sequences (> 350 bp) from this study compared with sequences available in GenBank. Sequences recovered from a single sample are sequentially numbered after the sample name. [file 42523_2021_114_MOESM4_ESM.docx]

**Table S4.** Nucleotide identity between partial L1 gene sequences (> 350 bp) from this study compared with sequences available in GenBank. Sequences recovered from a single sample are sequentially numbered after the sample name.

| **BPV type/putative new BPV type** | **Sequence identification** | **Sample** | **Best BLASTn hit/GenBank accession number** | **L1 nucleotide identity (%)** |
| --- | --- | --- | --- | --- |
| BPV25 | 4833RS16/BR-1 | 4833RS16/BR | BPV25 strain 14RS13/BR/ MG252779.1 | 99.74 |
| BPV7 | 4151RS16/BR-2 | 4151RS16/BR | BPV7 strain IT-221/ KM096429.1 | 99.36 |
| BPV7 | 4151RS16/BR-6 | 4151RS16/BR | BPV7 strain IT-221/ KM096429.1 | 99.25 |
| putative new BPV type | 3891RS16/BR-39 | 3891RS16/BR | BPV24 strain 06AC14/ MG602223.1 | 75.92 |
| putative new BPV type | 4150RS16/BR-9 | 4150RS16/BR | BPV17/ KU519392.1 | 74.36 |
| putative new BPV type | 3689RS16/BR-51 | 3689RS16/BR | BPV12 strain PR000002/ JF834524.1 | 72.22 |
| putative new BPV type | 3689RS16/BR-1 | 3689RS16/BR | BPV12 strain PR000002/ JF834524.1 | 72 |
| putative new BPV type | 4826RS16/BR-74 | 4826RS16/BR | RtiPV2 strain IZW 39/08/ NC_040785.1 | 66.79 |
| putative new BPV type | 3891RS16/BR-11 | 3891RS16/BR | RtiPV2 strain IZW 39/08/ NC_040785.1 | 66.25 |
