# Supplementary figures and images for: The genetic diversity of “papillomavirome” in bovine teat papilloma lesions
Source: Anim Microbiome. 2021 Jul 28;3:51. doi: 10.1186/s42523-021-00114-3 (PMC8317299; doi:10.1186/s42523-021-00114-3)

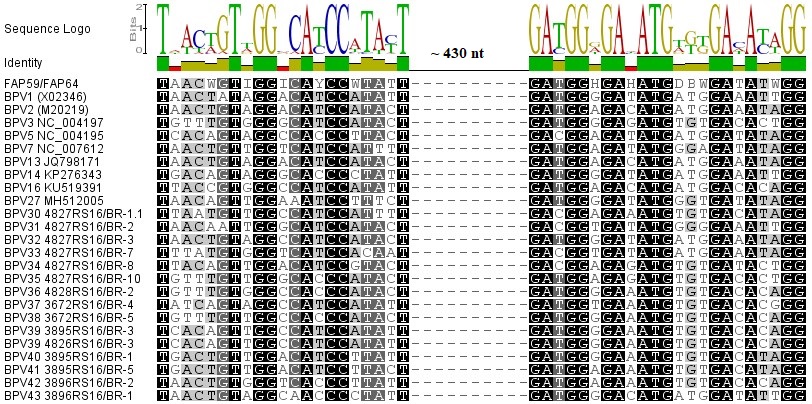

Supplement: Supplementary file 6 — Additional file 6. FAP primer pair annealing sites in all the new PV L1 sequences. Alignment of the BPV L1complete sequences demonstrating the annealing efficiency between the sequences of this study and classical BPVs (Delta, Xi, Epsilon, Dyoxi and Dyokappapapillomavirus genera). The color gradation shows the degree of nucleotide identity, where black represents 100% identity between the nucleotide sequences. In the upper part, the graph indicates a summary of the most frequent nucleotides. Nucleotide alignment was generated using MUSCLEaligner with default settings in Geneious software v. 9.0.5. [file 42523_2021_114_MOESM6_ESM.jpg]
